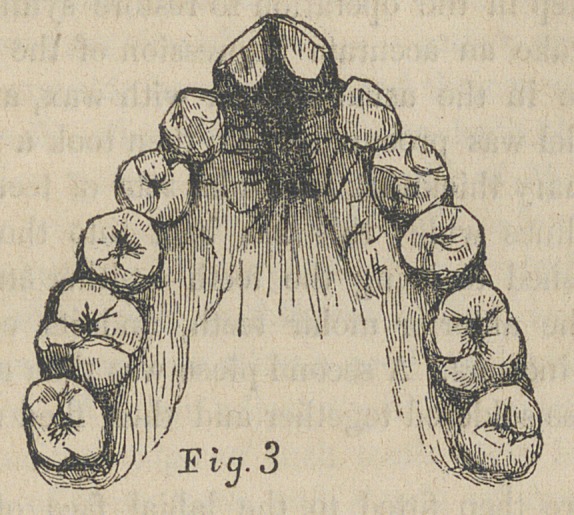# Method of Directing Second Dentition and Correcting Irregularity

**Published:** 1855-01

**Authors:** James Taylor


### Dental Register of the West

VOL. VIII. JANUARY, 1855. NO. 2.

### Original Communications.

### METHOD OF DIRECTING SECOND DENTITION AND CORRECTING IRREGULARITY.

BY JAMES TAYLOR, M.D., D.D.S.

Continued from page 9, vol. viii.

In our last we promised to give a few cases of irregularity, relieved by a bar and ligatures.

We give the most simple case first. It was merely an inclination inward of the right supeiioı central incisor. Miss C——— is about 22 or 23 years of age, and this tooth stood so far in as very much to disfigure an otherwise beautiful denture. Owing to the smallness of the necks of her teeth, the ligatures failed to act as finely as we desired. And owing to this fact, the ligatures *would slip* up under the gums and keep them constantly sore and irritable; and the teeth were too far apart to allow the cross ligatures to be used, for the knot at the approximate edges of the teeth would slip up and thus not keep to the proper place the main ligature. We might have used gold hooks for this purpose, but the teeth were not as firmly *socketed* as many, and we wished to draw inward and slightly towards the medial line the lateral incisor of the same side. We constructed a Bar represented in figure first. This bar is just long enough to allow its ends to rest on the cuspid teeth, and is so bent at the ends as to partially embrace their labial face.

Two small hooks are soldered to this bar, one of which hooks over the cutting edge of the left central incisor, and the other over the cutting edge of the right lateral incisor. They are made of small strips of ordinary gold plate. The bar is made of two strips of gold plate soldered together, first having been adjusted with plyers, to fit the front teeth, standing off of the irregular tooth, so as to permit traction to draw it to its proper place. The hooks keep the bar from slipping upon the gum. We thus secured the bar far enough below the gum, so that the ligature which passed around the irregular teeth and was tied to the bar, kept the thread from irritating the gum—a few days sufficed to bring the tooth to its place—the ligature should be removed at least every other day and be re-applied; this gives opportunity for cleansing the teeth and tightning the ligatures.

In such cases the bar and ligature should be kept on for one or two weeks after the irregular teeth are brought into place; unless this is done they will soon settle back into their old position.

The above kind of cases, we almost invariably treat with ligatures alone, and regard such cases as affording less trouble than any other.

*Fig. 3*

CASE II.—The position of the teeth in this case are not truly represented by figure third, for this is from an impression taken after the case had been under treatment some ten days or more. The first cast having been lost or broken, and the bicuspids had by this time been drawn almost half their thickness further out, into their proper circle.

The inferior bicuspids at first struck at each occlusion of the jaws outside of the superior ones, the under incisors coming in contact with the points of the two central incisors of the superior jaw; but owing to their obliquity, the medial half of these teeth are in a proper occlusion of the jaws outside of the lower teeth. The lateral incisors in the upper jaw had both been extracted; the right had been lost early in life, and owing to malformation and decay of the left we extracted this some year or two since. The cuspids of the superior jaw articulated inside of the lower teeth. The appearance of this mouth was a projecting chin and a short retracting upper lip. To remedy this deformity, and give a beautiful mouth—to an accomplished, interesting, and otherways fine looking young lady—was a matter of some moment. Miss———, whose case we have now under consideration, is about 17, teeth very firm, and constitutionally rather good. Her mother had from mal-formation of the superior lateral incisors lost both when young. *Query*,—how long will this hereditary disposition continue in the family?

The first step in the operation to restore symmetry in this case, was to take an accurate impression of the upper teeth; this was done in the usual manner with wax, and from this a plaster model was procured. We then took a piece of gold plate of ordinary thickness, for under sets of teeth, and about one to two lines wide; this was bent into the circle—into which we wished to bring the teeth—either end of this bar resting on the anterior molar teeth, and its center merely touching the incisors. A second piece was then made like the first, and these soldered together and then filed up to the size we wished.

Clasps were then fitted to the labial face of the molars, passing as far between these and the posterior bicuspids as was practicable. The bar was then soldered to these, as is represented in cut No. II. When applied, it is attached to the molars by the clasps, and circles from these around in the proper circle of the teeth, resting on the central incisors; the bicuspids and cuspids stood within this circle about the thickness of these teeth.

In the application of this fixture, a ligature made of double patent thread, waxed, was passed around each of the displaced teeth, and fastened by a single tie. Then each end of the ligature was brought over the bar and tied fast, making that amount of traction desired. These ligatures were renewed every other day, until the teeth were brought to their proper place. We had progressed thus far in the operation, and had kept the teeth in this position a week or ten days, when Miss——— started East, expecting to be absent two or three months. Previous to leaving, we took an impression of these teeth and the alveolar arch, and fitted up a stiff gold plate, extending back to the molars, and neatly adjusted to the necks of the bicuspids and cuspids on either side. A clasp was fitted to the lingual face of each molar, and soldered fast to this plate. When adjusted to the mouth, it kept these teeth to their place, and was to have been worn until the teeth became firm in their place. It was worn about two weeks, being removed daily and cleansed. The plate was then thrown aside. A few days since Miss——— called, and we find that the teeth on one side have maintained their proper place. The bicuspids on the other side have fallen back about one-fourth of the position gained, and the cuspid of the same side a little, but its point still sticks outside of the lower teeth. We are now turning into proper position the central incisors.

This we accomplish as follows:—First wedge the teeth apart sufficiently wide to allow a good sized gold wire to pass between them. We then adjust a gold bar, extending from the lateral approximal edge of each tooth; this is fitted to the lingual face of these teeth, and stands off from them at the medial line; a hole is made in the bar parallel with this line, and a gold wire inserted and soldered fast. This wire passes between the teeth, and is sufficiently long to pass through a gold bar which is adjusted to their labial face; on the end of this wire is cut a screw, and for this a tap of thick gold plate is made. When this fixture is applied, the tap or nut is screwed on until it is drawn tight on the teeth; and every day or every other day screwed up a little tighter. Each day brings the two gold bars nearer together, and hence turns these teeth in their sockets. When these are turned to the point we want, we shall again adjust the plate to the inside of the teeth, and by wedging against the bicuspids which have dropped a little in, force them back and then hold all to their place until they become fixed.

We may be asked, what time is consumed in such an operation? We would say from two to three months—this case requiring more time, because interrupted before it was finished.

We have had several cases of the kind above described, some much worse; but have given this, because we happen to have the fixtures on hand, from which to get our cuts.

We are aware that several questions suggest themselves in this treatment. First, will not the pressure made on the molar teeth, by the tightening of the ligatures move these teeth? We answer, such has not been the case. If it should, we would fit the plate inside, and apply this, at the same time making pressure against these teeth.

Second, are the front teeth pressed in by the bar which rests upon them? We answer not, for unless the molar gives, the bar is too stiff to diminish its own circle by all the traction needed.

Third, does not the bar slip up on the gum? We answer, that where the teeth are very close, it can be tied down by threads passed between them and over the bar, and then tied at the points of the teeth, and when this is impracticable, two or three hooks can be soldered fast to the bar and made to catch over the points of some of the teeth, as in cut No. 1.

When one or more of the upper teeth strike inside of the lower, their antagonist teeth are capped and an inclined plane soldered on to strike these until they pass out—having thus indeed a double traction exerted upon them—which soon brings them in place. This enables us also to draw back or forward at this time any teeth which articulate on the wrong side of their antagonists.

We regard the proper articulation of the teeth as of the greatest importance, and unless due attention is paid to this in the treatment of irregularity, much trouble will often be met with, which otherwise would be avoided. The lower cuspid tooth for instance, which at every occlusion of the jaws strikes the posterior bevel of the outer cusp of the superior anterior bicuspid, must continue to keep this tooth pressed against the cuspid, and thus not permit it to fall back so as to give room for the incisors. This is sometimes even true when the posterior bicuspid has been removed; thus preventing that approximation of the anterior bicuspid and molar which is desired. A proper examination, and the teeth all being sound, but this articulation existing, would often point out the removal of the anterior instead of the posterior bicuspid. Should disease however between the molar and bicuspid shew itself, the loss of this would he demanded, and then the use of such artificial means as is necessary to keep the teeth apart while the change of articulation is effected.

We had a case a year or two since, where from the loss of the lateral incisors of the superior jaw, the articulation of the bicuspids on both sides, also cuspids, had to be changed. These teeth also stood their full thickness within their proper circle and stiking entirely within the lower. In the proper treatment of such cases, the change which is effected in the form of the palital arch is often remarkable. Instead of having a narrow contracted arch, impeding very much articulation, it is flattened out, giving far more room to the tongue and often entirely remedying that lisping which is so observable in many of these cases. This alone is of such importance, and the benefit so palpable, that it far more than compensates the patient for all the pain and trouble attendant upon such cases, even when they are of the most protracted kind.

Another great desideratum is the proper use of the teeth in mastication. In such cases the occlusion of the jaws is generally such, that the teeth are almost useless as *grinding* organs. There is no adaptation, and hence no opportunity for a proper comminution of the food.

We have been delighted to see, how that after the teeth have been once gotten into their proper place, how gradually and yet how surely the cusps glide into their appropriate places when the mouth is shut. A year may sometimes be not more than sufficient for this, even after all our work is done; and all this time an improvement is taking place in the appearance of the teeth, as well as in their use.

Having thus arranged the teeth, we let nature finish the operation.

## Figures and Tables

**Fig. 2 f1:**
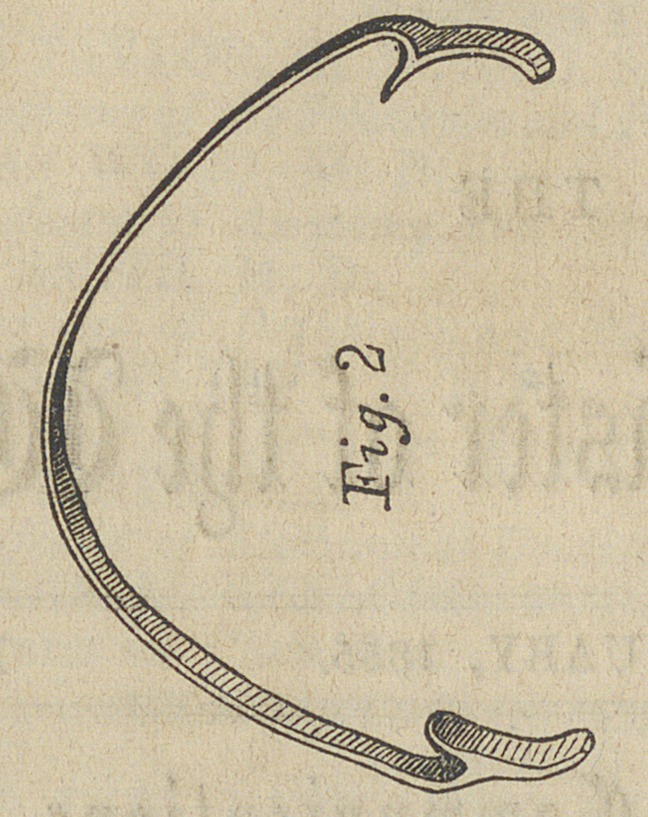


**Fig. 1 f2:**
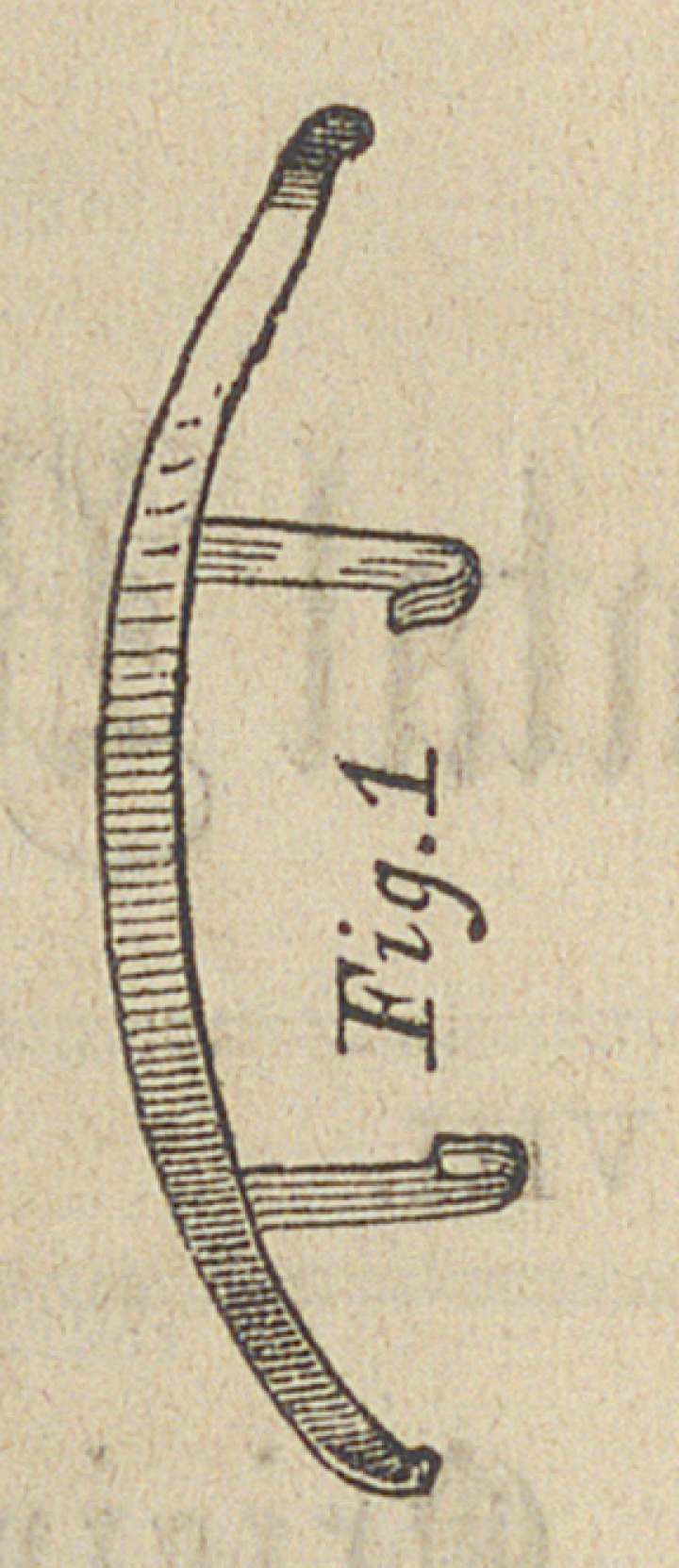


**Fig. 3 f3:**